# Distance to food stores & adolescent male fruit and vegetable consumption: mediation effects

**DOI:** 10.1186/1479-5868-4-35

**Published:** 2007-09-13

**Authors:** Russell Jago, Tom Baranowski, Janice C Baranowski, Karen W Cullen, Debbe Thompson

**Affiliations:** 1 Department of Exercise, Nutrition & Health Sciences,University of Bristol, Tyndall Avenue, Bristol, BS8 1TP, UK; 2 USDA/ARS Children's Nutrition Research Center, Department ofPediatrics, Baylor College of Medicine, 1100 Bates Street, Houston, TX 77030-2600, USA

## Abstract

**Background:**

The physical environments in which adolescents reside and their access to food stores may influence their consumption of fruit and vegetables. This association could either be direct or mediated via psychosocial variables or home availability of fruit and vegetables. A greater understanding of these associations would aide the design of new interventions. The purpose of this study was to examine associations between distance to food stores and restaurants and fruit and vegetable consumption and the possible mediating role of psychosocial variables and home availability.

**Methods:**

Fruit and vegetable consumption of 204 Boy Scouts was assessed by a food frequency questionnaire in 2003. Participant addresses were geo-coded and distance to different types of food stores and restaurants calculated. Fruit and vegetable preferences, home availability and self-efficacy were measured. Regression models were run with backward deletion of non-significant environmental and psychosocial variables. Mediation tests were performed.

**Results:**

Residing further away from a small food store (SFS) (convenience store and drug store) was associated with increased fruit and juice and low fat vegetable consumption. Residing closer to a fast food restaurant was associated with increased high fat vegetable and fruit and juice consumption. Vegetable preferences partially mediated (26%) the relationship between low fat vegetable consumption and distance to the nearest SFS.

**Conclusion:**

Distance to SFS and fast food restaurants were associated with fruit and vegetable consumption among male adolescents. Vegetable preferences partially mediated the distance to low fat vegetable relationship. More research is needed to elucidate how environmental variables impact children's dietary intake.

## Background

Fruit and vegetable consumption has been associated with a decreased risk of several forms of cancer [1,2] and reduced risk of obesity [3]. Many children [4,5] in the USA do not consume five servings of fruit and vegetables per day. Current behavioral models explain a relatively small percentage of the variance in children's fruit and vegetable consumption [6]. Improved understanding of the factors that influence youth fruit and vegetable consumption and how they interact is needed to guide intervention design [7].

Psychological theories enhance our ability to understand behavior by identifying key mediators of the behavior of interest that can subsequently be manipulated in an intervention [8]. Social cognitive theory [9] has been used extensively to design youth fruit and vegetable interventions [10-14]. A key feature of SCT is reciprocal determinism, the notion that patterns of behaviour are dynamic, constantly being affected by both characteristics and beliefs of the individual and the environment in which the behaviour is performed [9,15]. Thus, while a number of studies have reported that youth fruit and vegetable consumption differ according to the age [16], gender [16], ethnicity [17] and socio-economic status [17] of the participants, a great deal of attention has also focused on psychosocial variables that could be manipulated in an intervention. Fruit and vegetable self efficacy [18] (perceived competence to select and eat fruit and vegetables) and preferences (the extent to which the child likes fruit and vegetables [7,19,20]) are two psychosocial variables consistent with SCT that have been shown to predict youth fruit and vegetable consumption.

Reciprocal determinism also specifies that environmental variables interact with participant psychosocial variables to influence behavior. Home availability of fruit and vegetables (whether fruit and vegetables are available to be eaten in the home) is a proximal environmental variable that has consistently predicted consumption [21]. There is also evidence that broader environmental factors influence fruit and vegetable consumption. For example, among White and Black Americans fruit and vegetables consumption increased with access to supermarkets [22], while reduced access to grocery stores was associated with lower diet quality among pregnant women [23]. The availability of fruit, fruit juice and vegetables at restaurants within the census tract in which Boy Scouts resided was also associated with fruit juice and vegetable consumption [24].

**Figure 1:**
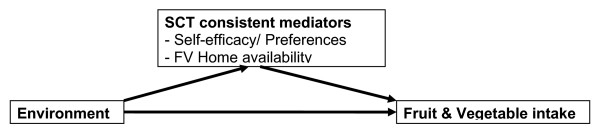
Hypothesized SCT mediation model to be tested.

A limitation of existing research has been the consideration of just psychosocial or only environmental influences on fruit and vegetable consumption. Social cognitive theory suggests that the association between environmental variables and behavior could be either direct or indirect (e.g. a facilitating or buffering effect) [25]. An indirect association would provide further support for SCT as it would suggest that the association is mediated by other variables such as psychosocial variables or home availability of fruits and vegetables, which is a more proximal environmental variable [26]. These associations are shown in Figure 1. In this paper we test the hypothesis that the relationship between environmental variables and adolescent fruit and vegetables consumption is mediated by psychosocial variables or home availability. We assessed whether: 1) distance to the nearest/density of food outlets was associated with adolescent male fruit and vegetable consumption; 2) associations between fruit and vegetable consumption and environmental features change after accounting for psychosocial variables and home availability; and 3) psychosocial variables or home availability mediated the association between the fruit and vegetable consumption and distance to or density of food outlets.

## Methods

Participants were 204 Boy Scouts (aged 10–14) recruited from 36 Boy Scout Troops within the greater Houston (TX) area with a mean of 5.8 participants per troop (range 2 – 12). Participants were a sub-sample from the baseline assessment of a physical activity intervention [27]. Due to a shortage of accelerometers in the main intervention study, accelerometer data were only collected on a randomly selected sub-sample. The addresses of participants that met a minimum two-day accelerometer inclusion criteria were geo-coded and associations with environmental characteristics reported [28,29]. A series of tests indicated no differences in the BMI (t = .983, df = 449, p = 0.326), age (t = 1.33, df = 466, p = 0.894), fruit, juice or vegetable intake (t = .282, df = 448, p = 0.778) or parental education (χ^2 ^= 2.65, df = 3, p = 0.448) between these participants and the larger sample. The Baylor College of Medicine Institutional Review Board approved this study and written informed consent and assent were obtained for all participants.

Parental report provided participant ethnicity and place of residence. As socioeconomic status can vary within census tract and participants came from many census tracts within Houston we used the highest education obtained within the household as a family specific indicator of socioeconomic status. As over 70% of the participants were Euro-American, ethnicity was coded as Euro-American or Other. Stature was measured to the nearest 0.1 cm using a stadiometer (Shorr Height Measuring Board; Olney, MD) and weight was measured to the nearest 0.1 kg using a calibrated scale (Seca 770 Model Scale; Vogel and Halke, Hamburg). Body mass index (BMI = kg/m^2^) and BMI percentile were computed [30].

### Dietary assessment

Fruit, juice and vegetable consumption were assessed using the Cullen Food Frequency Questionnaire [31] which assesses consumption of 4 juices, 17 fruits and 17 vegetables. The scale had a test re-test Spearman correlation of 0.54 among Boy Scouts [31]. In the US 100% juice is an approved component of the US 5 A Day program [32]. Consumption of juice is usually low and so it was combined with fruit servings because evidence suggests that youth view fruit and juice separately from vegetables [33]. Separate analyses were conducted for fruit and 100% juice and vegetable consumption. Fruit and juice consumption was computed by summing servings of the 4 100% juices and 17 fruits. Two separate vegetable variables were created. The first category was high fat vegetables which included fried potatoes (French Fries) as well as potato salad and coleslaw which were included as in Texas they are usually made using high fat mayonnaise. The second category was low fat vegetables (the remaining 14 vegetables).

### Ecological and psychosocial variables

Fruit and vegetable home availability was assessed using the GEMS scale which had good internal consistency (Cronbachs alpha = 0.77) and reasonable 12-week test re-test reliability (ICC = 0.50) [34]. Items were summed to provide the home availability of fruit and juice, low fat vegetables and high fat vegetables. Fruit juice and vegetable preferences were assessed for the same items using the Domel scale which has internal consistencies ranging from 0.67 to 0.93 and test-retest reliabilities of 0.69 to 0.83 [35]. Self-efficacy was assessed using the Domel scale which had high internal consistency (Cronbachs alpha > 0.72) [36]. The scale included statements about both fruit and vegetables together and as such it was therefore not possible to create separate scales. Consequently, a combined self-efficacy score was used in the analyses. Social desirability was measured through the administration of the "lie scale" from the revised Manifest Anxiety Scale to control for socially desirable responses [37].

### Environmental assessment

The environmental assessment has been reported elsewhere [29]. Briefly, each participant's home address was geo-coded using ArcGIS Version 9.0 (ESRI, Redlands, California). The ArcView buffer tool was used to create a boundary with a 1-mile radius (1609.3 m) around each participant's residence. One-mile is the upper limit of commonly used interpretations of neighborhood walk-ability [29,38-41]. City council public health records were used to identify the number of food establishments within each participant's buffer zone. Grocery stores were grouped according to the following North American Industry Classification System 2002 codes [42]: supermarket (445110); small food store, which included the food stores other than large supermarkets which sell food and included convenience stores (445120) and drug stores (446110); meat, fish, vegetable or fruit (4452100, 445220, 445230) and warehouse club (452910). Restaurants were grouped using the following codes: full service restaurant (772110), cafeteria (722212) and fast food restaurant (722211). This process provided the number or density of food outlets within a 1-mile radius of the participant's home address. The Euclidean (crow-flies) distance to the nearest of each of these categories of stores was also calculated using the ArcGIS software. Although some investigators have used the street network distance many youths do not follow street patterns, they use informal paths (such as routes across vacant lots) and as such the crow-flies distance provides a standard, replicable measure [29,43].

### Statistics

Frequency distributions, means and standard deviations were calculated. Linear regression models that controlled for the clustering of participants within Boy Scout troops were performed using the ***xtreg ***procedure in STATA (Version 9.0, College Station, TX). All regression models included troop as a random effect and predictors as fixed effects. Three separate analyses were run with either fruit and juice, high fat vegetable or low fat vegetable consumption as the dependent variable.

Models to test for main effects and possible mediation were run in five steps. To ensure that relationships of interest were not due to confounding variables, step one included personal characteristics (BMI percentile, age, parental education and ethnicity). As children's reports of fruit and vegetable consumption have been influenced by social desirability [44] this was also included in step one. The remaining four steps facilitated the testing of predictors and mediation effects using the criteria of Baron and Kenny [45]. In step two, demographics were retained and all of the nearest environmental variables were entered into the model. Non-significant environmental variables (p > .06) were removed in a step-wise backward deletion process. (This step assessed the criterion that environmental variables were correlated with the dependent variable). Step three included all of the demographic variables from step one plus preferences, self-efficacy and home availability and non-significant variables were backward deleted, (thereby testing the criterion that the mediator is associated with the dependent variable). In step four, regression models were run (which also controlled for demographics) to ascertain whether environmental variables were associated with psychosocial variables, the hypothesized mediators, (thereby testing the criterion that the predictor is associated with the mediator). Step five included demographics plus significant environmental and psychosocial variables from steps two and three with non-significant variables backward deleted. If these four criteria were met, a Sobel test was performed using the STATA ***sgmediation ***command to determine the extent of mediation. The within group and between group associated R^2 ^values for each regression model were obtained as was the overall R^2 ^which is comparable to the adjusted R^2 ^obtained from non-clustered models.

The process was then repeated using the density of each type of food outlet. **As none of these variables were significant (p > .06) **the models are not reported.

## Results

Participants were 12.8 years of age with a mean BMI percentile of 62.8 (Table 1). Over 70% of the participants had a parent who received a college education. The mean daily intake of FJV was 5.9 servings per day.

**Table 1:** Participant characteristics

**Variable**	**N**	**Mean**	**SD**
BMI	197	21.0	4.7
BMI %tile	195	62.8	30.7
Age	208	12.8	1.1
			
**Ethnicity**	N	%	
Euro-American	146	70.2	
Other	62	29.8	
			
**Parental Education (N = 208)**	N	%	
GED	15	7.2	
Tech College	44	21.2	
College	70	33.7	
Postgraduate	79	38.0	
			
**Diet**	**N**	**Mean**	**SD**
Fruit (svgs)	204	2.3	2.7
Juice (svgs)	204	.9	1.0
Fruit and Juice (svgs)	200	3.1	2.7
Vegetables (svgs)	200	2.6	2.1
Low Fat Vegetables (svgs)	200	2.3	1.9
High Fat Vegetables (svgs)	200	.4	.5
Fruit, Juice and Vegetables (svgs)	200	5.9	5.4
			
**Psychosocial Variables**			
Social Desirability	202	30.7	7.0
Availability of Fruit & Juice	204	8.9	4.3
Availability of Vegetables	204	7.7	3.7
Availability of low fat vegetables	204	7.0	3.4
Availability of high fat vegetables	204	0.7	.88
Fruit Preferences	204	45.5	8.1
Vegetable Preferences	204	34.2	7.1
Low fat vegetable preferences	204	31.7	7.2
High fat vegetables preferences	204	2.4	.7
Fruit & Vegetable Self-Efficacy	204	82.0	15.7

There was an average of 1.3 supermarkets, 7.3 small food stores, 8.9 fast food restaurants and 9 full service restaurants within 1 mile radius of participants' addresses (Table 2). The average distance to the nearest small food store was 778 meters while the average distance to the nearest fast food restaurant was 1051 meters.

**Table 2:** Descriptive statistics for environmental variables

**Variable**	**N**	**Mean**	**SD**
**# within a 1 mile radius**			
Supermarket (SM)	210	1.3	1.5
Small Food Store (SFS)	210	7.3	6.3
Meat, Fish, Vegetable, Fruit Stall (MFVF)	210	.5	.9
Warehouse clubs (WC)	210	.1	.1
Fast Food restaurant (FF)	210	8.9	10.4
Cafeteria restaurant (CF)	210	.2	.5
Full service restaurant (FSR)	210	9.0	13.7
			
**Distance to the nearest (m)**			
Supermarket (SM)	210	1961.7	1871.7
Small Food Store (SFS)	210	777.9	625.5
Meat, Fish, Vegetable, Fruit Stall (MFVF)	210	3486.7	2735.5
Warehouse clubs (WC)	210	3320.2	2045.3
Fast Food restaurant (FF)	210	1051.3	869.8
Cafeteria restaurant (CF)	210	4628.9	4001.8
Full service restaurant (FSR)	210	1040.4	773.0

Regression models predicting fruit and juice consumption are shown in Table 3. In the first step, none of the personal characteristics were significant predictors of fruit and juice intake and the model accounted for a low proportion of the variance (less than 6%). In step two, distance to the nearest small food store was positively associated with fruit and juice consumption (Beta = 0.001 (95% CI = 0.00, 0.00), z = 3.07, p = 0.002) while distance to the nearest fast food restaurant was negatively associated (Beta = -0.000, (95% CI = -0.001, -0.000), z = -2.76 p = 0.006). The variance accounted for increased to 11% overall. In step three both fruit and juice home availability (Beta = 0.269 (95% CI = 0.18, 0.35), z = 6.37, p < 0.001) and preferences (Beta = 0.061 (95% CI = 0.02, 0.10), z = 2.80 p = 0.005) were associated with fruit and juice consumption. The addition of the psychosocial variables substantially increased the variance accounted for with the overall R^2 ^increasing to 25%. The mediation tests performed indicated that distance to the nearest small food store was associated with fruit and juice preferences (z = 2.17, p = 0.030), but not home availability. Distance to the nearest fast food restaurant was not associated with either fruit and juice preferences or home availability (p > 0.05). In step five, distance to the nearest small food store, distance to the nearest fast food restaurant, home availability and preferences were all (p < 0.05) associated with fruit and juice consumption.

**Table 3:** Regression models predicting fruit and juice intake

	**Step One (Personal characteristic**			**Step Two (Step 1 + nearest environmental variables)**			**Step Three (Step 1 + psychosocial variables)**			**Step Five (Step 1 + nearest environmental & psychosocial variables)**		
**Variable**	**Coeff)**	**Z**	**P**	**Coeff**	**Z**	**P**	**Coeff**	**Z**	**P**	**Coeff**	**Z**	**P**

**BMI %tile**	0.00	1.04	0.299	0.01	1.23	0.220	0.01	1.29	0.198	0.01	1.46	0.145
**Age**	-0.22	-1.24	0.216	-0.22	-1.26	0.207	-0.10	-0.65	0.516	-0.11	-0.72	0.474
**PG (ref GED)**	-0.35	-0.39	0.693	-0.07	-0.08	0.938	-0.69	-0.88	0.381	-0.47	-0.60	0.548
**College**	-0.99	-1.10	0.270	-0.76	-0.85	0.397	-1.22	-1.56	0.119	-1.04	-1.32	0.186
**Tech College**	-1.42	-1.49	0.135	-1.17	-1.26	0.209	-1.26	-1.51	0.130	-1.09	-1.33	0.182
**Ethnicity (ref Anglo)**	-0.49	-1.10	0.271	-0.72	-1.59	0.111	-0.38	-0.95	0.341	-1.39	-1.39	0.164
**Social Desirability**	0.03	1.12	0.262	0.03	1.09	0.277	0.02	0.94	0.347	0.03	0.97	0.332
**Distance to Small Food Store**	**-**	**-**	**-**	0.00	3.07	**0.002**		**-**	**-**	0.00	2.63	**0.008**
**Distance to Fast Food**	**-**	**-**	**-**	-0.00	-2.76	**0.006**		**-**	**-**	-0.00	-2.52	**0.012**
**FJ Home Availability**	**-**	**-**	**-**	**-**	**-**	**-**	0.27	6.37	**< 0.001**	0.27	6.38	**< 0.001**
**FJ Preferences**	**-**	**-**	**-**	**-**	**-**	**-**	0.06	2.80	**0.005**	0.05	2.31	**0.021**
**Within Troop R**^2^	**0.0468**			**0.0937**			**0.2495**			**0.2877**		
**Between Troop R**^2^	**0.0297**			**0.1317**			**0.5398**			**0.5380**		
**Overall R**^2^	**0.0523**			**0.1064**			**0.2485**			**0.3155**		

**Step Four**:

Distance to the nearest Small Food Store (SFS) was associated with FJ preferences (z = 2.17, p = 0.030) but not availability of fruit and juice (z = 0.04, p = 0.968).

Distance to the nearest fast food restaurant was not associated with FJ preferences (z = 0.35, p = 0.724) or FJ availability (z = -0.57, p = 0.570)

Sobel test indicated that Fruit and Juice preferences partially mediated (34%) the relationship between distance to nearest Small Food Store (SFS) and low fat vegetable intake (z = 1.805, p = 0.071)

Comparison of steps two and five indicates that the association between distance to the nearest small food store and fruit and juice consumption was attenuated (z reduced from 3.07 to 2.63) after preferences were added to the model suggesting the fruit and juice preferences functioned as a mediator. A Sobel test indicated that fruit and juice preferences partially mediated (34%) the association between fruit and juice intake and distance to the nearest small food store, but the mediation effect was not statistically significant (z = 1.805, p = 0.071).

Regression models predicting the consumption of low fat vegetables are shown in Table 4. None of the personal characteristics were significant predictors in step 1. In step 2, distance to the nearest small food store was associated with low fat vegetables consumption (Beta = 0.001, (95% CI = 0.00, 0.001), z = 2.74, p = 0.006). In step three, preferences (Beta = 0.067 (95% CI = 0.02, 0.09), z = 3.04, p = 0.002) and home availability (Beta = 0.182 (95% CI = 0.10, 0.26), p < 0.001) were positively associated with consumption. The addition of these psychosocial variables to the demographics also substantially increased the overall variance accounted by the model from less than 4% to greater than 21%. In step four, distance to the nearest small food store was associated with low fat vegetables preferences (z = 2.32, p = 0.020), but not home availability (z = 0.47, p = 0.638). In step five low fat vegetables preferences (Beta = 0.050 (95% CI = 0.1, 0.09), Z = 2.70 p = 0.007) and availability (= 0.177 (95% CI = 0.09, 0.25), p < 0.001) predicted consumption while distance to the nearest small food store approached significance (Beta = 0.003 (95% CI = -0.00, 0.00), z = 1.87, p = 0.060).

**Table 4:** Regression models predicting Low Fat Vegetable consumption

	**Step One (Personal characteristics)**			**Step Two (Step 1 + nearest environmental variables)**			**Step Three (Step 1 + psychosocial variables)**			**Step Five (Step 1 + nearest environmental & psychosocial variables)**		
**Variable**	**Coeff**	**Z**	**P**	**Coeff**	**Z**	**P**	**Std.**	**Z**	**P**	**Coeff**	**Z**	**P**

**BMI %tile**	0.003	0.73	0.464	0.003	0.77	0.441	0.003	0.81	0.418	0.003	0.85	0.394
**Age**	0.070	0.56	0.573	0.069	0.57	0.568	0.029	0.26	0.797	0.030	0.27	0.786
**PG (ref GED)**	0.593	0.97	0.330	0.900	1.48	0.139	0.423	0.76	0.444	0.635	1.13	0.257
**College (ref GED)**	0.042	0.07	0.946	0.348	0.57	0.569	-0.084	-0.15	0.880	0.129	0.23	0.819
**Tech College (ref GED)**	0.136	0.21	0.834	0.214	0.34	0.737	0.069	0.12	0.907	0.130	0.22	0.824
**Ethnicity (ref Anglo)**	0.338	1.09	0.276	0.205	0.67	0.506	0.211	0.75	0.453	0.131	0.47	0.641
**Social Desirability**	0.009	0.45	0.656	-0.002	-0.10	0.923	0.006	0.31	0.753	-0.001	-0.04	0.964
**Distance to Small Food Store**	**-**	**-**	**-**	0.001	2.74	**0.006**	**-**	**-**	**-**	0.000	1.87	**0.060**
**Low Fat Veg Preferences**	**-**	**-**	**-**	**-**	**-**	**-**	**0.057**	**3.04**	**0.002**	0.050	2.70	**0.007**
**Low Fat Veg Home Availability**	**-**	**-**	**-**	**-**	**-**	**-**	**0.181**	**4.58**	**< 0.001**	0.177	4.48	**< 0.001**
**Within Troop R**^2^	**0.0100**			**0.0447**			**0.1888**			**0.2130**		
**Between Troop R**^2^	**0.1193**			**0.3366**			**0.5985**			**0.6124**		
**Overall R**^2^	**0.0338**			**0.0741**			**0.2190**			**0.2348**		

**Step Four**: Distance to the nearest Small Food Store (SFS) was associated with low fat vegetable preferences **(**z = 2.32, p < 0.020) but **not **low fat vegetable home availability (z = 0.47, p = 0.638)

Sobel test indicated that low fat vegetable preferences partially mediated (26%) the relationship between distance to nearest Small Food Store (SFS) and low fat vegetable intake (z = 2.13, p = 0.032)

Reduction in the strength of association between distance to the nearest small food store and low fat vegetables consumption before and after the addition of low fat vegetables preferences (z reduced from 2.74 to 1.87) suggested a mediation effect. This hypothesis was supported by the Sobel test which indicated that low fat vegetable preferences partially mediated (26%) the relationship between distance to the nearest small food store and low fat vegetables intake (z = 2.13, p = 0.032).

Regression models predicting the consumption of high fat vegetables are shown in Table 5. None of the personal characteristics were significant in step one. In step two, distance to the nearest small food store was positively associated with high fat vegetable consumption (Beta = 0.003 (95% CI = 0.00, 0.00), z = 3.41, p < 0.001) while distance to the nearest fast food restaurant was negatively associated (Beta = -0.001, (95% CI = -0.00, -0.00), z = -2.94 p = 0.003). The model accounted for 8% of the variance within troops and 10% of the overall variance. In step three, home availability (Beta = 0.165 (95% CI = 0.07, 0.26), z = 3.60, p < 0.001) and preferences (Std. Beta = 0.174 (95% CI = 0.07, 0.28), z = 3.22 p = 0.001) were associated with consumption. In step four neither distance to the nearest small food store nor distance to the nearest fast food restaurant was associated with preferences or availability indicating no mediation effects. In step five, distance to the nearest small food store (Beta = 0.003 (95% CI = 0.00, 0.00), z = 3.69, p < 0.001), home availability (Beta = 0.169 (95% CI = 0.08, 0.26), z = 3.79, p < 0.001) and preferences (Beta = 0.174 (95% CI = 0.07, 0.27), z = 3.31, p = 0.001) were associated with consumption while distance to the nearest fast food restaurant was negatively associated (Beta = -0.001 (95% CI = -0.00, -0.00), z = -3.21, p = 0.001). The step five model accounted for 22.5% of the overall variance.

**Table 5:** Regression models predicting High Fat Vegetable consumption

	**Step One (Personal characteristics)**			**Step Two (Step 1 + nearest environmental variables)**			**Step Three (Step 1 + psychosocial variables)**			**Step Five (Step 1 + nearest environmental & psychosocial variables)**		
**Variable**	**Coeff**	**Z**	**P**	**Coeff**	**Z**	**P**	**Coeff**	**Z**	**P**	**Coeff**	**Z**	**P**

**BMI %tile**	0.001	1.03	0.303	0.002	1.25	0.210	0.002	1.57	0.117	0.002	1.83	0.067
**Age**	0.033	0.93	0.353	0.033	0.96	0.335	0.015	0.44	0.659	0.015	0.45	0.563
**PG (ref GED)**	-0.108	-0.62	0.536	-0.042	-0.24	0.807	0.012	0.07	0.942	0.089	0.55	0.585
**College (ref GED)**	-0.038	-0.22	0.826	0.018	0.10	0.917	0.055	0.33	0.740	0.117	0.72	0.473
**Tech College (ref GED)**	0.066	0.35	0.723	0.116	0.64	0.523	0.108	0.62	0.538	0.164	0.97	0.334
**Ethnicity (ref Anglo)**	-0.070	-0.79	0.428	-0.119	-1.36	0.173	0.021	0.25	0.805	-0.027	-0.32	0.748
**Social Desirability**	0.000	0.08	0.937	0.000	0.01	0.995	0.04	0.77	0.439	0.004	0.77	0.443
**Distance to Small Food Store**	**-**	**-**	**-**	0.003	**3.41**	**0.001**	**-**	**-**	**-**	0.003	3.69	**< .001**
**Distance to Fast Food**	**-**	**-**	**-**	-0.001	**-2.94**	**0.003**	**-**	**-**	**-**	-0.001	-3.21	**0.001**
**High Fat Veg Home Availability**	**-**	**-**	**-**	**-**	**-**	**-**	0.165	3.60	**< .001**	0.169	3.79	**< .001**
**High Fat Veg Preferences**	**-**	**-**	**-**	**-**	**-**	**-**	0.174	3.22	**0.001**	0.174	3.31	**0.001**
**Within Troop R**^2^	**0.0246**			**0.0853**			**0.1515**			**0.2255**		
**Between Troop R**^2^	**0.0489**			**0.1372**			**0.1330**			**0.1634**		
**Overall R**^2^	**0.0368**			**0.1014**			**0.1591**			**0.2253**		

**Step Four**:

Distance to the nearest Small Food Store (SFS) was **not **associated with HFV Preferences (z = -0.22, p = 0.821) or Availability (z = 0.08, p = 0.936).

Distance to the nearest fast food restaurant was **not **associated with HFV preferences (z = -0.12, p = 0.908) or Availability (z = 0.25, p = 0.803)

## Discussion

Distance to the nearest small food store was a positive predictor of fruit and juice, low fat vegetable and high fat vegetable consumption, but proximity to large food stores was not associated with any of the dietary variables. Theoretically, shopping behavior will be conditional on local supply and the catchments of local grocery stores. If this were true eating behavior would be a function of distance to a large grocery store as people would need to drive further to get to where they do their regular large shop. We did not find this association in our data, but we did show that fruit and vegetable consumption was inversely associated with access to small stores. Since small stores usually provide a limited supply of fruit and vegetable [46], reduced proximity to these stores may limit consumption of higher calorie foods which negatively impacts fruit, juice and vegetable consumption. Moreover, among adolescents, who do not drive, it may be that access to small stores is more important than access to the larger grocery stores that require car access.

Twenty six percent of the association between distance to the nearest small food store and low fat vegetable consumption was mediated by preferences. Although the data are cross-sectional, and therefore the ability to detect the true nature of associations is not possible, the results indicate that participants who lived further away from small grocery stores had increased preferences for fruit and vegetables. Thus, adolescents who have less access to the smaller food stores, which traditionally carry a wider variety of processed foods and less fresh fruit and vegetables are perhaps more likely to consume fruit and vegetables [at home or in other locations] and develop preferences for them. Moreover, this group of adolescents are perhaps less likely to visit small stores and buy processed foods. Alternatively, it may be the case that families that like fruit and vegetables elect to live in neighborhoods that are further away from small food stores.

Distance to the nearest fast food outlet was negatively associated with both fruit and juice intake and high fat vegetable consumption. As proximity to a fast food restaurant increased so did consumption of fruit and juice and high fat vegetables. This seems logical because high fat vegetables such as French Fries are sold at these restaurants. Thus, perhaps adolescents who live close to a fast-food restaurant are more likely to consume the high fat vegetables provided at these stores, a simple facilitating effect. The association with fruit and juice is more difficult to tease out, but it may be that this association is a function of the fruit and juice that these stores sell, or children mistakenly reporting fruit pies or fruit flavored beverages as fruit consumed.

Although distance to small food stores and fast food restaurants was associated with consumption, no other proximity indicator was significant, nor were any density measures. This finding is in contrast to the research which has shown that access to supermarkets is associated with adult fruit and vegetables consumption [22,23,47-50]. The cause of this disparity is not clear. However, no previous research has focused specifically on US "Sun-Belt" cities or male adolescents. Thus, while the results of this study appear at odds with earlier research this study provides key information about a previously understudied group.

There was a small (~4%) increase in the variance accounted for by the addition of environmental variables. Approximately 30% of the total variance in fruit and vegetable consumption was explained by personal characteristics, psychosocial and environmental variables. This is comparable to previous studies [51-53] that have not included environmental variables. Home availability was the strongest predictor of fruit and juice and low fat vegetable consumption, but it did not function as a mediator of distance to the nearest grocery stores, suggesting that strategies that focus directly on increasing home fruit, juice and vegetable availability will increase intake [21]. The greater influence of home availability suggests that increasing home availability may be an effective intervention approach for this group.

For fruit and juice as well as low fat vegetables, the amount of variance accounted for by the models was much greater between troops than within troops when psychosocial variables were included in the models. For example, in Table 3 the between troop variance was 13% when just personal characteristics and environmental variables were in the model but this increased to 54% when personal and psychosocial variables were in the model. This suggests that participant self-efficacy, preferences and home availability account for more of the variability between troops whereas participants in the same troop are exposed to similar influences and also impact the self-efficacy and preferences of each other. This finding therefore supports our decision to control for clustering effects and implies that understanding why psychosocial variables differ between clusters could be important for developing new, more effective intervention approaches.

The mean daily fruit and vegetable consumption of this sample was 5.9 servings per day which is comparable to the 5.2 servings per day that were previously reported among Houston Boy Scouts using the same instrument [54]. The 5.9 servings per day found in this study is greater than the 4.3 servings per day that were obtained using the BRFSS fruit and vegetable intake questionnaire among adolescent males in Minnesota [5]. As the physical activity and BMI profiles of this group are comparable to other studies of similar aged US adolescents [27] the sample does not appear to be an unusually "healthy group". Since food frequency questionnaires that assess intake of individual fruits and vegetables have been shown to yield higher estimates of fruit and vegetable intake [55] the relatively high levels reported in this study could be a by-product of the assessment method.

### Strengths and limitations

The strengths of this study are the combination of diet, psychosocial and objective environmental data. The use of a food frequency questionnaire which relies on the participants' perception of frequency and portion size [56] is a potential limitation of this study. Parental food decisions may be more important influences on childhood consumption than access to food stores, but we do not have data to examine this possibility. The predominantly Euro-American, relatively small male sample drawn from a single "sun-belt" city limits our ability to generalize to other groups including similar aged girls. It should also be noted that as our participants were predominately middle class the lack of variability is likely to have limited our ability to detect differences in association by socio-economic status which could be important in light of the previous work that has shown considerable economic [48-50] and ethnic [57] differences in access to grocery stores. Unfortunately, we do not have household income data which prevents us from fully exploring economic differences in the associations detected in our sample. We do, however, know that our sample was predominately middle class and predominately white. Therefore the null association between access to large grocery stores and fruit and vegetable consumption may be masked by a lack of variability in participant's socio-economic status. However, although the study has some limitations, given the shortage of information about the associations between dietary patterns, environmental characteristics and psychosocial variables and the absence of comparable data from adolescents this study provides data about an important area of public health research.

## Conclusion

Among adolescent males, residing further away from a small food store and close to a fast food restaurant was associated with increased fruit, juice and vegetable consumption. The association between distance to a small food store and low fat vegetable consumption was mediated by low fat vegetable preferences.

## Competing interests

The author(s) declare that they have no competing interests.

## Authors' contributions

This study was conceived by RJ and TB. RJ performed the analysis with all authors providing input on data interpretation. RJ wrote the first draft of the paper. All authors provided critical input and revisions to the paper. All authors approved the submission of this paper.

## References

[B1] Riboli E, Norat T (2003). Epidemiologic evidence of the protective effect of fruit and vegetables on cancer risk. Am J Clin Nutr.

[B2] Maynard M, Gunnell D, Emmett P, Frankel S, Davey Smith G (2003). Fruit, vegetables, and antioxidants in childhood and risk of adult cancer: the Boyd Orr cohort. J Epidemiol Community Health.

[B3] Epstein LH, Gordy CC, Raynor HA, Beddome M, Kilanowski CK, Paluch R (2001). Increasing fruit and vegetable intake and decreasing fat and sugar intake in families at risk for childhood obesity. Obesity research.

[B4] Brady LM, Lindquist CH, Herd SL, Goran MI (2000). Comparison of children's dietary intake patterns with US dietary guidelines. Br J Nutr.

[B5] Nystrom AA, Schmitz KH, Perry CL, Lytle LA, Neumark-Sztainer D (2005). The relationship of weight-related perceptions, goals, and behaviors with fruit and vegetable consumption in young adolescents. Prev Med.

[B6] Baranowski T (2006). Advances in basic behavioral research will make the most important contributions to effective dietary change programs at this time. J Am Diet Assoc.

[B7] Rasmussen M, Krolner R, Klepp KI, Lytle L, Brug J, Bere E, Due P (2006). Determinants of fruit and vegetable consumption among children and adolescents: a review of the literature. Part I: quantitative studies. Int J Behav Nutr Phys Act.

[B8] Baranowski T, Klesges LM, Cullen KW, Himes JH (2004). Measurement of outcomes, mediators, and moderators in behavioral obesity prevention research. Prev Med.

[B9] Bandura A (1986). Social foundations of thought and action: A social cognitive theory.

[B10] Baranowski T, Baranowski J, Cullen KW, deMoor C, Rittenberry L, Hebert D, Jones L (2002). 5 a day Achievement Badge for African-American Boy Scouts: pilot outcome results. Prev Med.

[B11] Baranowski T, Baranowski J, Cullen KW, Marsh T, Islam N, Zakeri I, Honess-Morreale L, deMoor C (2003). Squire's Quest! Dietary outcome evaluation of a multimedia game. American Journal of Preventive Medicine.

[B12] Baranowski T, Baranowski JC, Cullen KW, Thompson DI, Nicklas T, Zakeri IE, Rochon J (2003). The Fun, Food, and Fitness Project (FFFP): the Baylor GEMS pilot study. Ethn Dis.

[B13] Reynolds KD, Franklin FA, Binkley D, Raczynski JM, Harrington KF, Kirk KA, Person S (2000). Increasing the fruit and vegetable consumption of fourth-graders: results from the high 5 project. Preventive Medicine.

[B14] Reynolds KD, Yaroch AL, Franklin FA, Maloy J (2002). testing mediating variables in a school-based nutrition intervention program. Health Psychol.

[B15] Baranowski T, Perry CL, Parcel GS, Glanz K, Rimer BK, Lewis FM (2002). How individuals, environments, and health behavior interact: Social cognitive theory. Health behavior and health education: theory, research and practice.

[B16] Cooke LJ, Wardle J, Gibson EL, Sapochnik M, Sheiham A, Lawson M (2004). Demographic, familial and trait predictors of fruit and vegetable consumption by pre-school children. Public Health Nutr.

[B17] Melnik TA, Rhoades SJ, Wales KR, Cowell C, Wolfe WS (1998). Food consumption patterns of elementary schoolchildren in New York City. J Am Diet Assoc.

[B18] Kratt P, Reynolds K, Shewchuk R (2000). The role of availability as a moderator of family fruit and vegetable consumption. Health Educ Behav.

[B19] Blanchette L, Brug J (2005). Determinants of fruit and vegetable consumption among 6-12-year-old children and effective interventions to increase consumption. J Hum Nutr Diet.

[B20] Bere E, Klepp KI (2005). Changes in accessibility and preferences predict children's future fruit and vegetable intake. Int J Behav Nutr Phys Act.

[B21] Jago R, Baranowski T, Baranowski J (2007). Fruit & vegetable availability:  a micro environmental mediating variable?. Public Health Nutrition.

[B22] Morland K, Wing S, Diez Roux A (2002). The contextual effect of the local food environment on residents' diets: the atherosclerosis risk in communities study. Am J Public Health.

[B23] Laraia BA, Siega-Riz AM, Kaufman JS, Jones SJ (2004). Proximity of supermarkets is positively associated with diet quality index for pregnancy. Prev Med.

[B24] Edmonds J, Baranowski T, Baranowski J, Cullen KW, Myres D (2001). Ecological and socioeconomic correlates of fruit, juice, and vegetable consumption among African-American boys. Prev Med.

[B25] Kremers SP, de Bruijn GJ, Visscher TL, van Mechelen W, de Vries NK, Brug J (2006). Environmental influences on energy balance-related behaviors: A dual-process view. Int J Behav Nutr Phys Act.

[B26] Baranowski T, Lin LS, Wetter D, Resnicow K, Hearn MD (1997). Theory as mediating variables: Why aren't community interventions working as desired?. Annals of Epidemiology.

[B27] Jago R, Baranowski T, Baranowski J, Thompson D, Cullen K, Watson K, Liu Y (2006). Fit for life Boy Scout badge: Outcome Evaluation of a troop & internet intervention. Prev Med.

[B28] Jago R, Baranowski T, Baranowski JC (2006). Observed, GIS, and self-reported environmental features and adolescent physical activity. Am J Health Promot.

[B29] Jago R, Baranowski T, Harris M (2006). Relationships between GIS environmental features and adolescent male physical activity: GIS coding differences. Journal of Physical Activity and Health.

[B30] National Center for Health Statistics CDC Growth Charts: United States. http://www.cdc.gov/growthcharts/.

[B31] Cullen KW, Baranowski T, Baranowski J, Hebert D, Moor CD (1999). Pilot study of the validity and reliability of brief fruit, juice and vegetable screeners among inner city African American boys and 17 to 20 year old adults. J Am Coll Nutr.

[B32] Institute NC Five a Day for Better Health - NCI Monograph. http://www.pbhfoundation.org/pdfs/pulse/research/5adayresearch/NCImonograph.pdf.

[B33] Cullen KW, Baranowski T, Baranowski J, Warnecke C, de Moor C, Nwachokor A, Hajek RA, Jones LA (1998). "5 A Day" achievement badge for urban boy scouts: formative evaluation results. J Cancer Educ.

[B34] Cullen KW, Klesges LM, Sherwood NE, Baranowski T, Beech B, Pratt C, Zhou A, Rochon J (2004). Measurement characteristics of diet-related psychosocial questionnaires among African-American parents and their 8- to 10-year-old daughters: results from the Girls' health Enrichment Multi-site Studies. Prev Med.

[B35] Domel SB, Baranowski T, Davis H, Leonard SB, Riley P, Baranowski J (1993). Measuring fruit and vegetable preferences among 4th- and 5th-grade students. Prev Med.

[B36] Baranowski T, Thompson WO, Davis HC, Leonard SB, Baranowski J, Domel. S. B (1996). Psychosocial predictors of fruit and vegetable intake in children. Health Education Research, Theory and Practice.

[B37] Reynolds CR, Paget KD (1983). National normative and reliability data for the Revised Children's Manifest Anxiety Scale. School Psychol Rev.

[B38] Pikora TJ, Bull FCL, Jamrozik K, Knuiman M, Giles-corti B, Donovan RJ (2002). Developing a reliable audit instrument to measure the physical environment for physical activity. American Journal of Preventive Medicine.

[B39] Duncan M, Mummery K (2005). Psychosocial and environmental factors associated with physical activity among city dwellers in regional Queensland. Prev Med.

[B40] Frank LD, Andresen MA, Schmid TL (2004). Obesity relationships with community design, physical activity and time spent in cars. Am J Prev Med.

[B41] Braza M, Showmaker W, Seeley A (2004). Neighborhood design and rates of walking and biking to elementary school in 34 California communities. Am J Health Promot.

[B42] US Census Bureau North American Industry Classification System (NAICS). http://www.census.gov/epcd/www/naics.html.

[B43] Forsyth A, Schmitz KH, Oakes M, Zimmerman J, Koepp J (2006). Standards for environmental measurement using GIS: Toward a protocol for protocols. Journal of Physical Activity and Health.

[B44] Klesges LM, Baranowski T, Beech B, Cullen K, Murray DM, Rochon J, Pratt C (2004). Social desirability bias in self-reported dietary, physical activity and weight concerns measures in 8- to 10-year-old African-American girls: results from the Girls Health Enrichment Multisite Studies (GEMS). Prev Med.

[B45] Barron RM, Kenny D (1986). The moderator-mediator variable distinction in social psychological research: conceptual, strategic, and statistical considerations. Journal of Personality and Social Psychology.

[B46] Zenk SN, Schulz AJ, Hollis-Neely T, Campbell RT, Holmes N, Watkins G, Nwankwo R, Odoms-Young A (2005). Fruit and vegetable intake in African Americans income and store characteristics. American Journal of Preventive Medicine.

[B47] Rose D, Richards R (2004). Food store access and household fruit and vegetable use among participants in the US Food Stamp Program. Public Health Nutr.

[B48] Kamphuis CBM, van Lenthe FJ, Giskes K, Brug J, Mackenbach JP (2006). Percieved environmental determinants of physical activity and fruit and vegetable consumption among high and low socioeconomic groups in the Netherlands. Health and Place.

[B49] Turrell G, Blakely T, Patterson C, Oldenburg B (2004). A multilevel analysis of socioeconomic (small area) differences in household food purchasing behaviour. J Epidemiol Community Health.

[B50] Moore LV, Diez Roux AV (2006). Associations of neighborhood characteristics with the location and type of food stores. American Journal of Public Health.

[B51] Wind M, De Bourdeaudhuij I, te Velde SJ, Sandvik C, Due P, Klepp KI, Brug J (2006). Correlates of Fruit and Vegetable consumption among 11-year-old Belgian-Flemish and Dutch schoolchildren. J Nutr Educ Behav.

[B52] Martens MK, van Assema P, Brug J (2005). Why do adolescents eat what they eat? Personal and social environmental predictors of fruit, snack and breakfast consumption among 12-14 year old Dutch adolescents. Public Health Nutr.

[B53] Baranowski T, Cullen KW, Baranowski J (1999). Psychosocial correlates of dietary intake: advancing dietary intervention. Annu Rev Nutr.

[B54] Cullen KW, Baranowski T, Baranowski J, Hebert D, de Moor C (1999). Pilot study of the validity and reliability of brief fruit, juice and vegetable screeners among inner city African-American boys and 17 to 20 year old adults. J Am Coll Nutr.

[B55] Krebs-Smith SM, Heimendinger J, Subar AF, Patterson BH, Pivonka E (1995). Using food frequency questionnaires to estimate fruit and vegetable intake: Association between the number of questions and total intake. J Nutr Educ.

[B56] Willett WC (1998). Nutritional Epidemiology.

[B57] Zenk SN, Schulz AJ, Israel BA, James SA, Bao S, Wilson ML (2005). Neighborhood racial composition, neighborhood poverty, and the spatial accessibility of supermarkets in metropolitan Detroit. American Journal of Public Health.

